# Evaluation of the relationship between plasma glucagon-like peptide-2 and gastrointestinal dysbiosis in canine chronic enteropathies

**DOI:** 10.1371/journal.pone.0305711

**Published:** 2024-06-27

**Authors:** Caylie D. Voudren, Erin J. Mayhue, Michelle D. Riehm, Maria C. Jugan

**Affiliations:** Department of Clinical Sciences, College of Veterinary Medicine, Kansas State University, Manhattan, KS, United States of America; Washington State University - Spokane, UNITED STATES

## Abstract

Chronic enteropathies are a common cause of morbidity in dogs and are associated with disruption of the normal gastrointestinal mucosal barrier. The objective of this prospective study was to determine the association between measures of gastrointestinal dysbiosis and plasma concentrations of glucagon-like peptide-2, a hormone responsible for normal mucosal structure, in dogs with chronic enteropathies. Fecal 16S V4 rRNA gene sequencing and quantitative PCR via the dysbiosis index was performed on 16 healthy controls and 18 dogs with chronic enteropathy prior to and 1 month after initiation of individualized therapy. Fasting and post-prandial plasma GLP-2 concentrations were measured via ELISA in healthy dogs and chronic enteropathy dogs at both time points. Alpha and beta diversity indices, as well as bacterial population abundances were compared between groups and time-points. Principal component analysis combined with least squares regression was used to identify taxa contributing to glucagon-like peptide-2 variance among groups. While the dysbiosis index did not differ between healthy dogs and dogs with chronic enteropathy, 16S V4 genomic sequencing identified 47 operational taxonomic units that differed between the groups, all but 2 of which resolved following chronic enteropathy treatment. Principal component analysis identified 6 families and 19 genera that contributed to differences in glucagon-like peptide-2 concentrations between groups. Dysbiosis associated with chronic enteropathies in dogs may contribute to the observed lower plasma glucagon-like peptide-2 concentrations. Further research into mechanisms of microbiota impact on the enteroendocrine system is needed. Association between glucagon-like peptide-2 secretion and microbiome indices may help to guide research into future treatment strategies for dogs with chronic enteropathy.

## Introduction

Chronic idiopathic enteropathy (CE) is a common disease of dogs typically classified by response to treatment (e.g., diet-responsive, immunosuppressant-responsive); although, its true pathogenesis is poorly characterized. The underlying etiology is likely multifactorial, including genetic predisposition, dietary and environmental influences, aberrant host response to luminal antigens and normal microbiota, and gastrointestinal (GI) dysbiosis [1]. Histopathologic changes to the GI tract of CE dogs include villus stunting, inflammatory infiltration of the lamina propria, and ultrastructural changes to the GI brush border, which aids nutrient digestion and absorption [2–4]. As GI inflammation is strongly associated with increased permeability to antigens, exploring mechanisms of decreased mucosal barrier function and maintenance in CE dogs may prove beneficial in development of novel therapeutic options.

In addition to inflammatory changes to the GI tract, GI microbiome dysbiosis and metabolome disturbances (e.g., fecal short chain fatty acid [SCFA] and bile acid composition) are consistently documented in dogs with CE [5–10]. Glucagon-like peptide-2 (GLP-2) is a post-translational peptide cleavage product of proglucagon that is normally secreted by enteroendocrine L-cells. The predominant role of GLP-2 is to maintain the GI mucosal barrier and normal GI structure, effects which are mediated through increased GI epithelial cell proliferation paired with decreased apoptosis, enhanced mucosal barrier function, and decreased mucosal proinflammatory cytokine expression [11–13]. While intraluminal nutrients provide the primary stimulus for GLP-2 secretion from L-cells, GI bacterial metabolic by-products, such as SCFA and bile acids, as well as normal microbiota, also stimulate L-cell secretion [14, 15]. Further, in rodents with GI hyperpermeability, prebiotic supplementation led to similar GI structural improvements as those seen with GLP-2 treatment, and correlations between GLP-2 concentrations and specific GI bacterial populations were noted [12, 16–20]. Initial investigation of microbiota impact on GLP-2 focused on individual bacterial species [16–18]; although, recent findings in humans with ulcerative colitis (UC) suggest that decreased circulating GLP-2 is associated with a general decrease in fecal microbiota diversity and abundances versus specific bacterial population shifts [21].

Variable changes in GLP-2 secretion have been documented in humans with CE (e.g., Crohn’s disease, UC) [22–24]. Under normal circumstances, circulating GLP-2 concentrations exhibit a rapid post-prandial increase in healthy humans [25]. In multiple human studies assessing GLP-2 secretion in adult and pediatric patients with Crohn’s disease, a post-prandial increase was not observed; although, fasting GLP-2 concentrations did not differ from healthy individuals [22, 26, 27]. In pediatric human patients with ileal Crohn’s disease, decreased post-prandial circulating GLP-2 concentrations normalized with disease response, and normalization of serum GLP-2 concentration was associated with enhanced mucosal surface area and function, suggesting that loss contributed to the observed decrease in GLP-2 [22]. However, a blunted post-prandial GLP-2 response was persistent in adult human patients in remission from Crohn’s disease [26, 27]. Similarly, decreased circulating GLP-2 concentrations were recently documented in dogs with CE compared to healthy dogs [28]. Serum GLP-2 concentrations increased following 1 month of standard treatment; although, measured concentrations still differed from the healthy dogs [28].

Given the association between GI microbiota and metabolic by-products that contribute to enteroendocrine responses, it is possible that GI dysbiosis in CE dogs contributes to changes in GLP-2 secretion. Therefore, the objective of this study was to characterize an association between the GI microbiome and circulating GLP-2 in dogs with idiopathic CE. It was hypothesized that dysbiosis would be associated with lower GLP-2 concentrations and that microbiota associated with SCFA production would be related to higher circulating GLP-2.

## Materials and methods

### Study population

This study included fecal samples from 16 healthy control (HC) dogs and 18 dogs with CE recruited prospectively between March 1, 2021, and May 26, 2022, as part of a previously conducted study with informed owner consent, which was obtained in writing at the time of enrollment [28]. The CE population included 9 dogs with either lymphoplasmacytic enteritis or gastroenteritis, 3 dogs with eosinophilic enteritis, 3 dogs with histiocytic or granulomatous enteritis, 2 dogs with undefined disease (i.e., no histopathology performed), and 1 dog with neutrophilic enteritis. Lymphoplasmacytic colitis was diagnosed in two dogs with concurrent small intestinal inflammatory disease. Inclusion criteria for the CE group followed standard protocols to exclude systemic disease and overt neoplastic or infectious enteropathies, including abdominal ultrasound performed by a board-certified veterinary radiologist in all dogs [28]. Testing for specific infectious etiologies (e.g, histoplasmosis), endocrine disease (e.g., hypoadrenocorticism), or pancreatic disease was performed at the discretion of the clinician managing the dog’s case. Trypsin-like immunoreactivity was evaluated in 10 dogs. Healthy control dogs were adult dogs with a body condition score (BCS) 4–6 out of 9 [29], normal baseline blood work, and no history of GI disease or any medications aside from routine heartworm, flea, and tick preventatives within the previous six months. Neither group had received raw food or raw food treats in the 6 months prior to study enrollment. When possible, a two-week trial with at least one novel protein or hydrolyzed diet was recommended prior to pursuing endoscopy; however, this was not a requirement of enrollment due to lack of some dogs’ willingness to eat a single diet.

### Experimental design

In all dogs, pre-prandial whole blood samples were collected for plasma GLP-2 measurement following a 10–15 hour fast. Blood sample handling was as previously described to prevent *in vitro* GLP-2 degradation [24, 28]. Fresh fecal samples were collected for microbiome analysis and dysbiosis index (DI). CE dog treatments were not standardized but rather determined by the clinician managing the dog’s case (S1 Table). Approximately 30 days after starting targeted CE therapy, study procedures and sample collection were repeated in the CE population. The CE population was divided into pre-treatment (CE-PRE) and post-treatment (CE-POST) groups for analysis. The Kansas State University IACUC approved all study procedures (Protocol 4479).

### Plasma GLP-2 measurement

A commercially available canine GLP-2 competitive ELISA kit (Canine GLP-2 ELISA Kit; Kendall Scientific) was used to measure plasma GLP-2 concentrations, as previously described [28]. In brief, after allowing plasma to thaw at room temperature for one hour, samples were analyzed in duplicate following manufacturer instructions. Fifty microliters of plasma were added per sample well (i.e., 100 μL total with duplication). A microplate reader (BioTek Epoch) was used to determine optical density at a wavelength of 450 nm immediately after the addition of stop reagent.

### Fecal sample collection, handling, and storage

Fecal samples analyzed in this study were collected at the time of defecation, immediately refrigerated at 4.5–5°C, stored at -80°C within 12 hours of defecation, and shipped on dry ice for DI (Texas A&M Gastrointestinal Laboratory) and comprehensive fecal microbiome analysis (Microbiome Insights). Samples were stored for <6–24 months prior to analysis.

### Fecal dysbiosis index

The fecal DI was calculated following quantitative PCR (qPCR) for total bacteria and specific bacterial taxa (i.e., *Faecalibacterium*, *Turicibacter*, *Streptococcus*, *Escherichia coli*, *Blautia*, *Fusobacterium*, and *Clostridium hiranonis*) according to standard laboratory protocol as a commercially available test (Texas A&M Gastrointestinal Laboratory). The DI was calculated using a previously described algorithm defining differences between healthy dogs and dogs with CE [9]. Consistent with previous work, a DI <0 was considered normal and >2 considered dysbiosis [9].

### Fecal DNA extraction and 16S rRNA gene sequencing

Fecal DNA extraction and amplicon sequencing based on 16S V4 rRNA (Illumina MiSeq) was performed by Microbiome Insights in a College of American Pathologists accredited laboratory. Fecal samples were placed into a MoBio PowerMag Soil DNA Isolation Bead Plate, and DNA was extracted using a KingFisher robot per manufacturer instructions. Bacterial 16S rRNA genes were PCR-amplified with dual-barcoded primers targeting the V4 region (515F 5’-GTGCCAGCMGCCGCGGTAA-3’ and 806R 5’-GGACTACHVGGGTWTCTAAT-3’) [30]. Amplicons were sequenced with an Illumina MiSeq using the 300-bp paired-end kit (v.3). Sequences were denoised, taxonomically classified using Silva (v. 138) as the reference database, and clustered into 97%-similarity operational taxonomic units (OTUs) (Mothur software package v. 1.44.1) [31]. The OTUs were then classified into taxonomic assignments. Sequencing quality was determined using FastQC 0.11.5 prior to classification and subsequent analysis.

Template-free negative controls were co-sequenced with DNA amplified from samples using the same procedures to assess for possible contamination. A positive control from ‘S00Z1-’ samples consisting of cloned SUP05 DNA, was also included. An OTU was considered a contaminant and removed from analysis if the mean abundance in controls reached or exceeded 25% of the mean abundance in samples.

### Statistical analysis

#### Dysbiosis index and qPCR

Data were tested for normality using the Shapiro-Wilk test; non-parametric analyses were used where data were not normally distributed. The Wilcoxon matched-paired signed-ranks test was used to compare the DI and logDNA of individual bacterial species between CE-PRE and CE-POST. The Mann-Whitney U test was used to compare the DI and logDNA of individual bacterial species between CE-PRE and HC. Bonferroni correction for multiple comparisons resulted in a *P* value of <0.003 for significance. Analyses were performed using commercial statistical software (GraphPad Prism v10.1.0).

#### Fecal DNA extraction and 16S rRNA gene sequencing

Statistical analysis was performed by Microbiome Insights. An analytical flowchart is included in supplementary material (S1 Fig). Alpha diversity was estimated with the Shannon index on raw OTU abundance tables. Shannon diversity was compared among groups (HC, CE-PRE, CE-POST) using an ANOVA, accounting for repeated measures and subsequent pairwise testing. To estimate beta diversity across samples, Bray-Curtis indices were computed after excluding OTUs with a count of less than 3 in at least 10% of the samples. Beta diversity was visualized using principal coordinate analysis (PCoA) ordination, emphasizing global differences in fecal microbial communities across samples. Variation in community structure was assessed with permutational multivariate analyses of variance (PERMANOVA) using treatment group as the main fixed factor and using 999 permutations for significance testing. Post-hoc pairwise testing was performed with FDR method correction for multiple comparisons. The *Linda* function from MicrobiomeStat was used to identify differentially abundant taxa using a linear model on centered log ratio transformed data. All analyses were conducted in the R environment (Version 4.1.2).

#### GLP-2 and microbiome comparison

Analyses were performed using commercially available statistical software (GraphPad Prism v10.1.0). Principal component analysis was performed at the phyla, family, and genus levels on taxa identified as differentially abundant in the above analyses and logDNA of taxa included in DI analyses. A least squares regression model with GLP-2 concentration as the dependent (outcome) variable and parallel analysis as the component selection method was used. Regression coefficients were converted to the scale of the original variable. A value of *P* < 0.05 was considered significant.

## Results

### Evaluation of the fecal microbiome

A total of 46 fecal samples [16 samples from HC dogs and 30 samples from a total of 18 CE dogs, 16 pre-treatment (CE-PRE) and 14 at follow-up (CE-POST)] were utilized for Illumina sequencing and qPCR assays. Of the 18 CE dogs, 2 dogs only had post-treatment fecal samples analyzed due to inadequate fecal sampling at baseline, and 4 dogs had only pre-treatment fecal samples analyzed due to study drop-out. The remaining dogs had both pre- and post-treatment samples analyzed. Illumina sequencing yielded a total of 1,282,880 sequences with an average of 19,935 quality-filtered reads per sample. The resulting dataset had 1760 OTUs, including singleton, which were divided into various taxa. A total of 6 phyla and 52 genera were identified (S2 Fig).

### Fecal microbiome in HC, CE-PRE, and CE-POST dogs

#### Dysbiosis index and qPCR

After accounting for multiple comparisons, there was no difference in the overall dysbiosis index between HC (median -6.10, 95% CI [-7.1,0.4]) and CE dogs at study enrollment (median -1.55, 95% CI [-4.50,3.90]; *P* = 0.016). Fecal *Turicibacter spp*. abundance was higher in HC (median logDNA 6.70, 95% CI [6.30,7.30]) compared to CE dogs at study enrollment (median logDNA 5.25, 95% CI [4.90,5.70]; *P* = 0.001). There were no other differences in fecal bacterial abundances via qPCR between HC versus CE-PRE in the DI or any fecal bacterial abundances between CE-PRE versus CE-POST (Table 1).

**Table 1:** Dysbiosis index and qPCR.

	Chronic Enteropathy		Healthy Control	*P* value	
	PRE	POST		CE^1^ PRE vs POST	CE^1^ PRE vs HC
Dysbiosis index	-1.55 (-4.50,3.90)	2.10 (-1.90,4.2)	-6.10 (-7.1,0.4)	0.123	0.016
**Bacteria (Log DNA)**					
*Blautia spp*.	10.05 (9.80,10.30)	9.70 (8.70,10.2)	10.10 (9.80, 10.20)	0.029	0.873
*C*. *hiranonis*	5.95 (0.40,6.40)	5.60 (0.10,6.20)	6.10 (5.90, 6.20)	0.309	0.582
*E*. *coli*	7.10 (4.40,7.80)	6.90 (5.30,7.40)	3.45 (2.10,6.20)	0.577	0.031
*Faecalibacterium spp*.	5.35 (4.80,6.90)	5.00 (4.80,5.90)	6.90 (6.30,7.30)	0.309	0.052
*Fusobacterium spp*.	8.55 (8.10,9.80)	8.30 (8.00,9.50)	9.20 (8.80, 9.80)	0.117	0.043
*Streptococcus spp*.	3.70 (2.60,4.90)	4.50 (3.40,7.60)	3.45 (2.60,5.90)	0.106	0.508
*Turicibacter spp*.	5.25 (4.90,5.70)	5.10 (4.80,6.10)	6.70 (6.30,7.30)	0.510	**0.001**

Median and 95% confidence interval of dysbiosis index and logDNA bacterial abundances in 16 healthy dogs, 16 untreated CE dogs (CE-PRE) and 14 CE dogs after 30 days of treatment (CE-POST). Bolded *P* values denote significance at *P* <0.003

^1^ CE = chronic enteropathy

### 16S V4 rRNA sequencing

**Fig 1:**
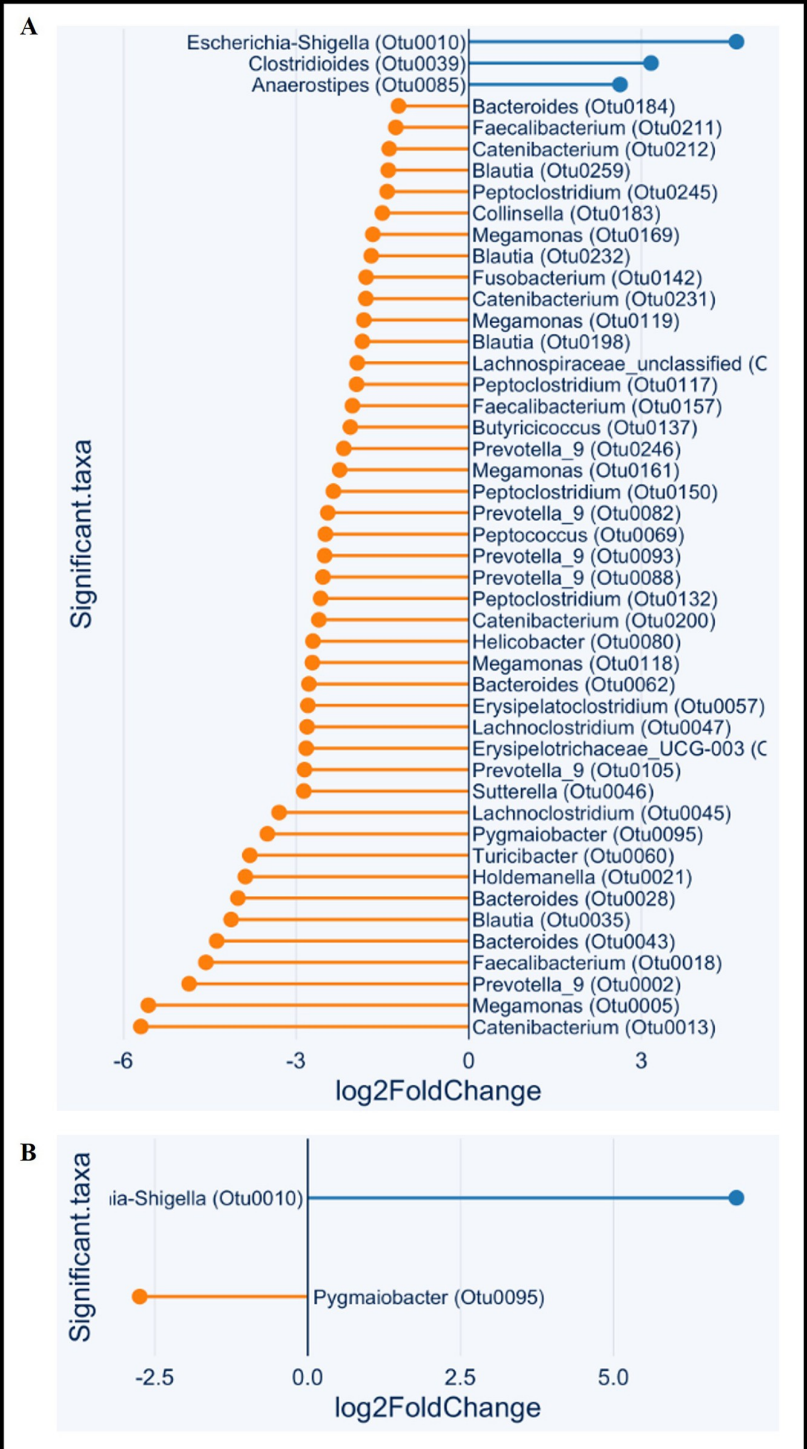
Fecal 16S V4 rRNA differential abundance testing in dogs with and without chronic gastrointestinal disease. Panel **(A)** represents the log fold changes in OTU differential abundances identified by 16S V4 rRNA genomic sequencing as significantly different between 16 dogs with untreated chronic enteropathy and 16 healthy controls. Panel **(B)** represents significantly different OTU abundances (log fold change) in 14 chronic enteropathy dogs after 30 days of individualized gastrointestinal disease treatment (CE-POST) and healthy controls. Each bar denotes either degree of increase (blue) or decrease (orange) of OTU differential abundance in the chronic enteropathy group compared to controls.

Differential abundance analysis pre-/post- intervention revealed 47 OTU which differed significantly between CE-PRE dogs compared to HC. All but three OTU within the genera *Anaerostipes*, *Clostridioides*, *Escherichia-Shigella* exhibited a relative decrease in abundance in CE dogs compared to HC. At follow-up, only 2 OTU significantly differed in abundance from HC in CE dogs, including a relative decrease in *Pygmaiobacter* and a relative increase in *Escherichia-Shigella* (Fig 1; S2 Table).

### Diversity indices

**Fig 2:**
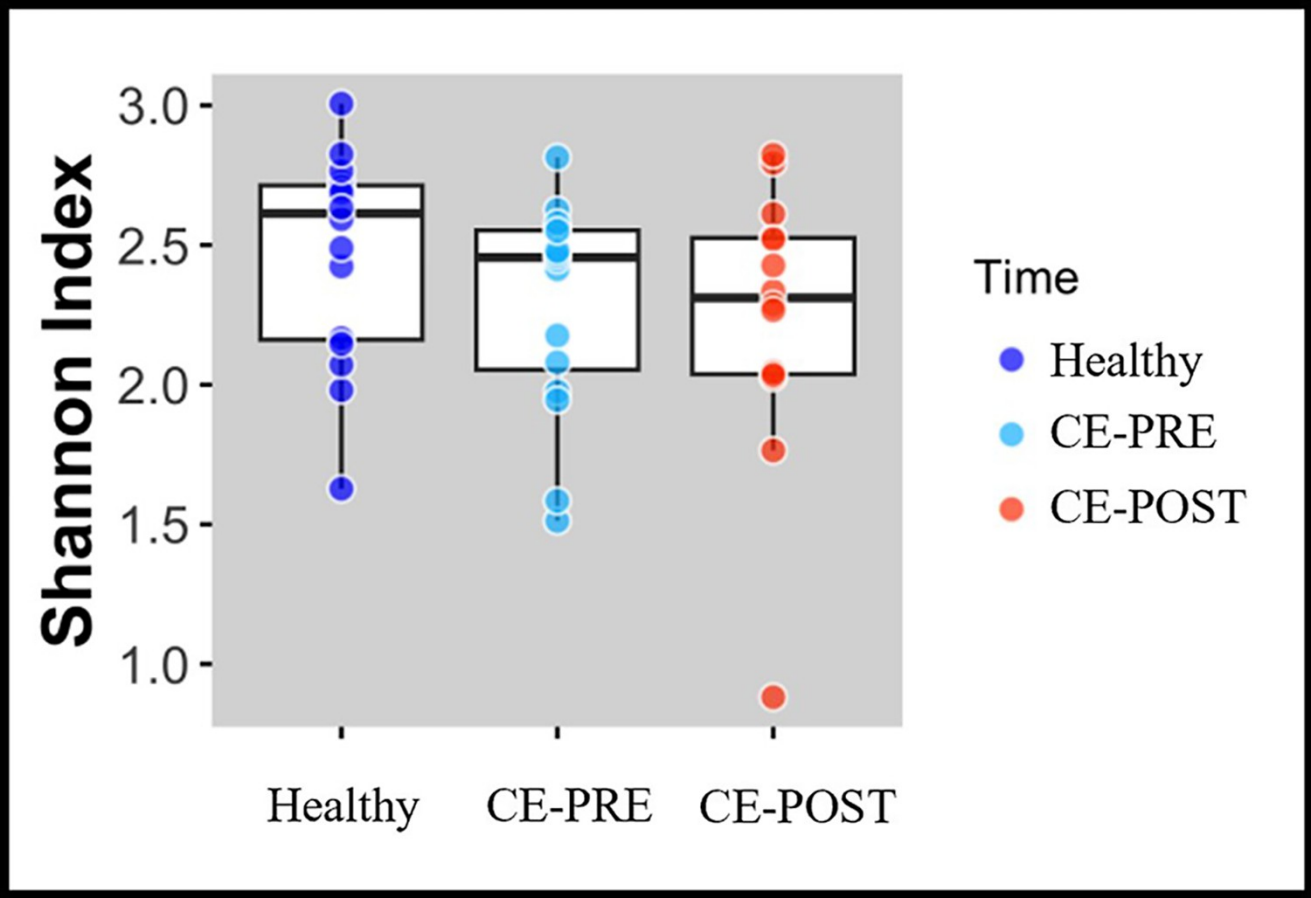
Boxplot of fecal microbiome Shannon diversity indices does not demonstrate differences between healthy dogs and dogs with chronic enteropathies. Mean and standard deviation Shannon index value estimates of fecal alpha diversity as measured by 16S V4 rRNA genomic sequencing in 16 healthy control dogs (HC), 16 untreated chronic enteropathy (CE-PRE) dogs and 14 CE dogs after 30 days of individualized gastrointestinal disease treatment (CE-POST). No significant differences (*P* > 0.05) were noted between groups.

**Fig 3:**
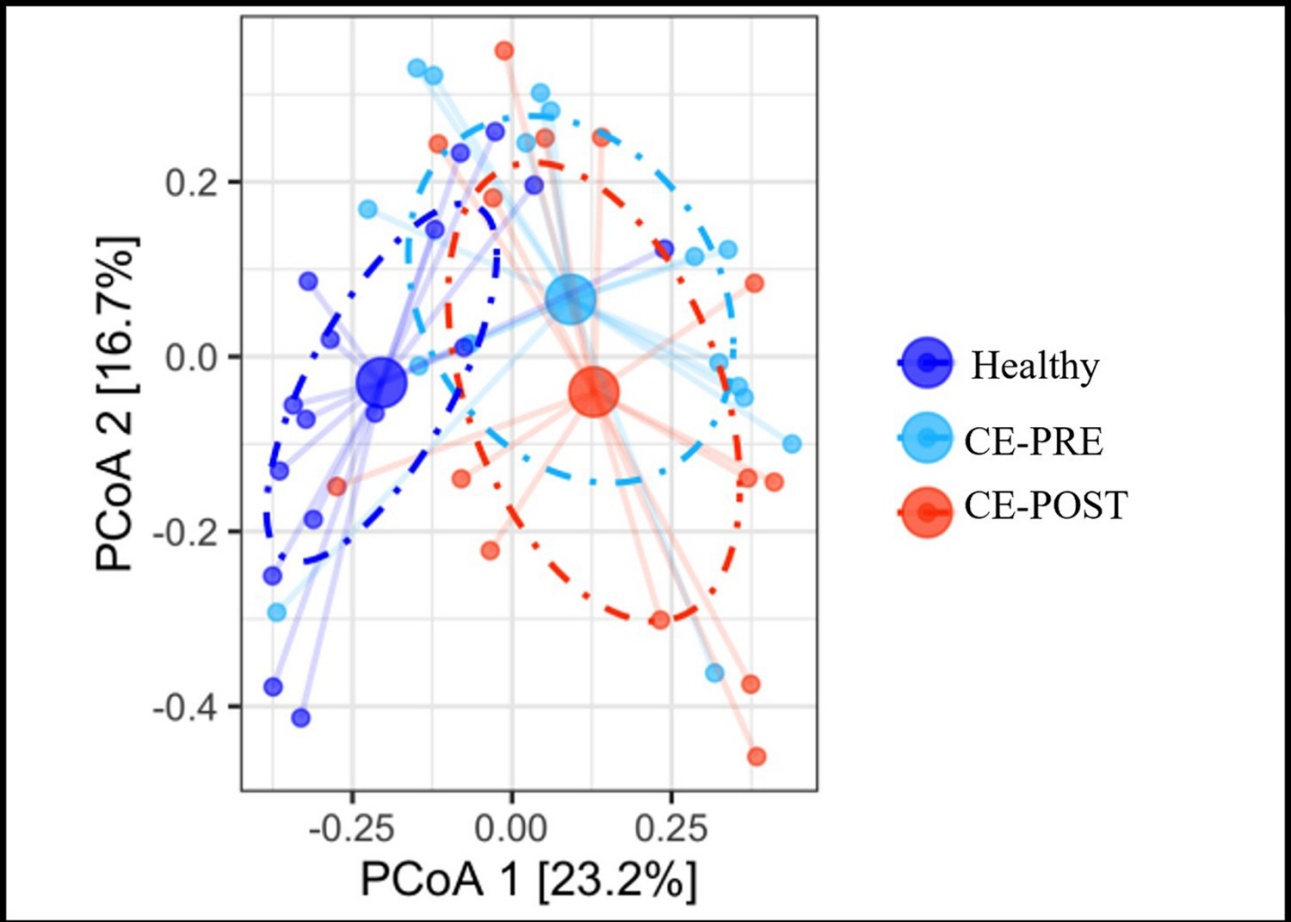
Fecal microbiome Bray-Curtis dissimilarities differentiate dogs with chronic enteropathies from healthy dogs Principal coordinate analysis (PCoA) ordination of Bray-Curtis dissimilarities of OTU in fecal samples of 16 healthy dogs compared to 16 dogs with untreated chronic enteropathies (CE-PRE) and 14 CE dogs after 30 days of individualized gastrointestinal disease treatment (CE-POST). Adonis testing was performed (R^2^ = 0.116; *P* **=** 0.001).

No significant differences were observed in alpha diversity, as described by the Shannon diversity index, when comparing HC or CE dogs at either timepoint (F 1.336; *P* = 0.274) (Fig 2). Compared to HC, CE dogs showed greater inter-individual distance in microbial community structure at both timepoints (*P* = 0.002). When compared to each other however, CE-PRE and CE-POST dogs displayed no significant differences in beta-diversity (*P* = 0.434) (Fig 3).

#### CE-associated microbiome shifts and GLP-2 concentrations

**Fig 4:**
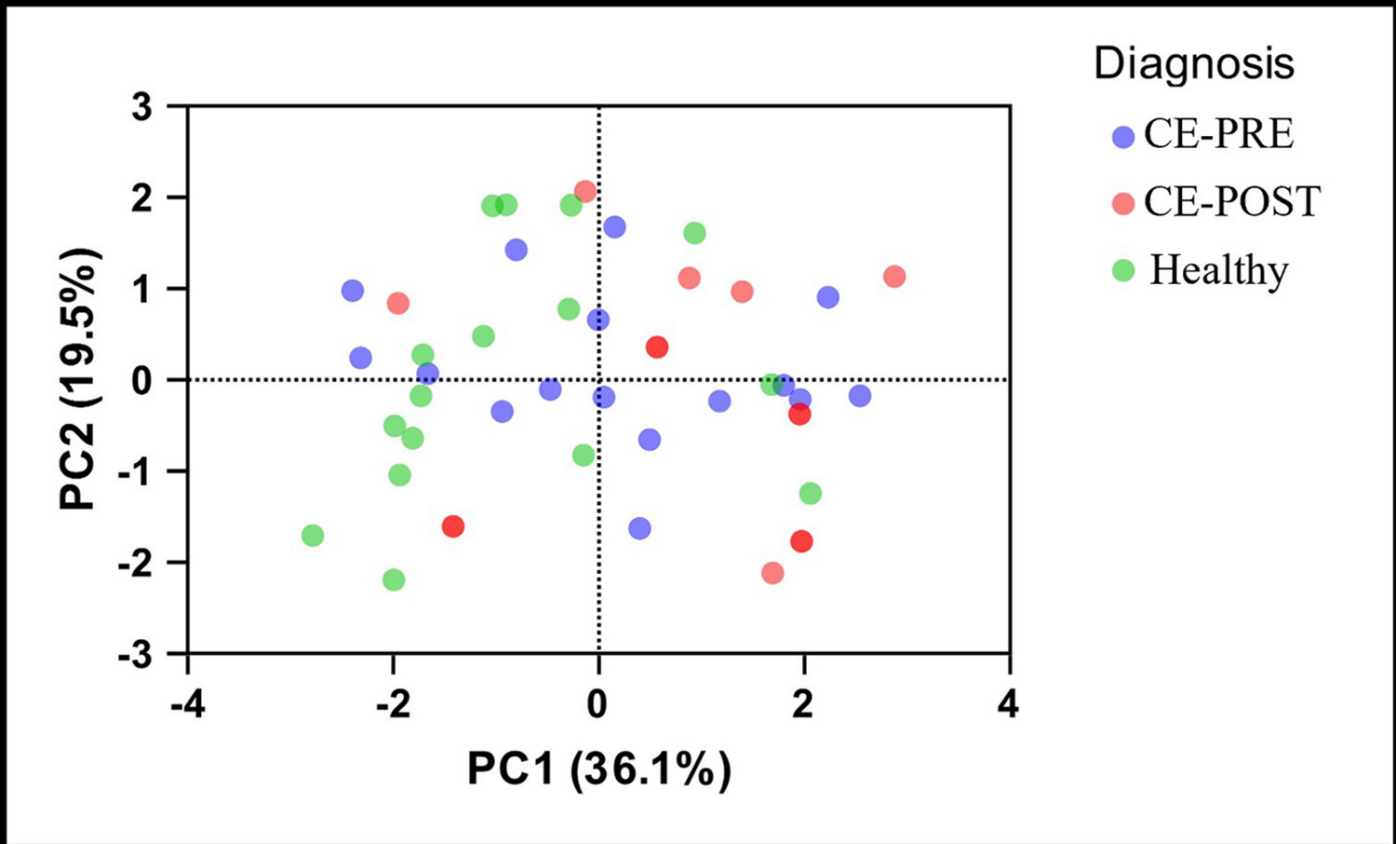
Principal component analysis (PCA) of the fecal microbiome using qPCR based on disease status. Fecal bacterial abundances (logDNA) detected by qPCR through dysbiosis index analysis were used to detect differences between 16 healthy dogs (green) compared to 16 dogs with untreated chronic enteropathies (CE-PRE; blue) and 14 CE dogs after 30 days of individualized gastrointestinal disease treatment (CE-POST; red). A least squares regression model performed on PCA with GLP-2 concentration as the outcome did not identify any taxa with significant contribution to GLP-2 variance.

No components of the DI explained the variance in GLP-2 (R^2^ = 0.157; F (7,38) = 1.007; *P* = 0.442) (Fig 4). When evaluating microbiome effects on GLP-2 concentrations based on 16S V4 rRNA analysis, phyla level analysis did not significantly explain variance in GLP-2 (R^2^ = 0.071; F (1,44) = 3.348; *P* = 0.074). Family level analysis explained 14.2% variance with PC1 and 11.22% variance with PC2 (R^2^ = 0.319; F (5, 40) = 3.741; *P* = 0.007).

**Fig 5:**
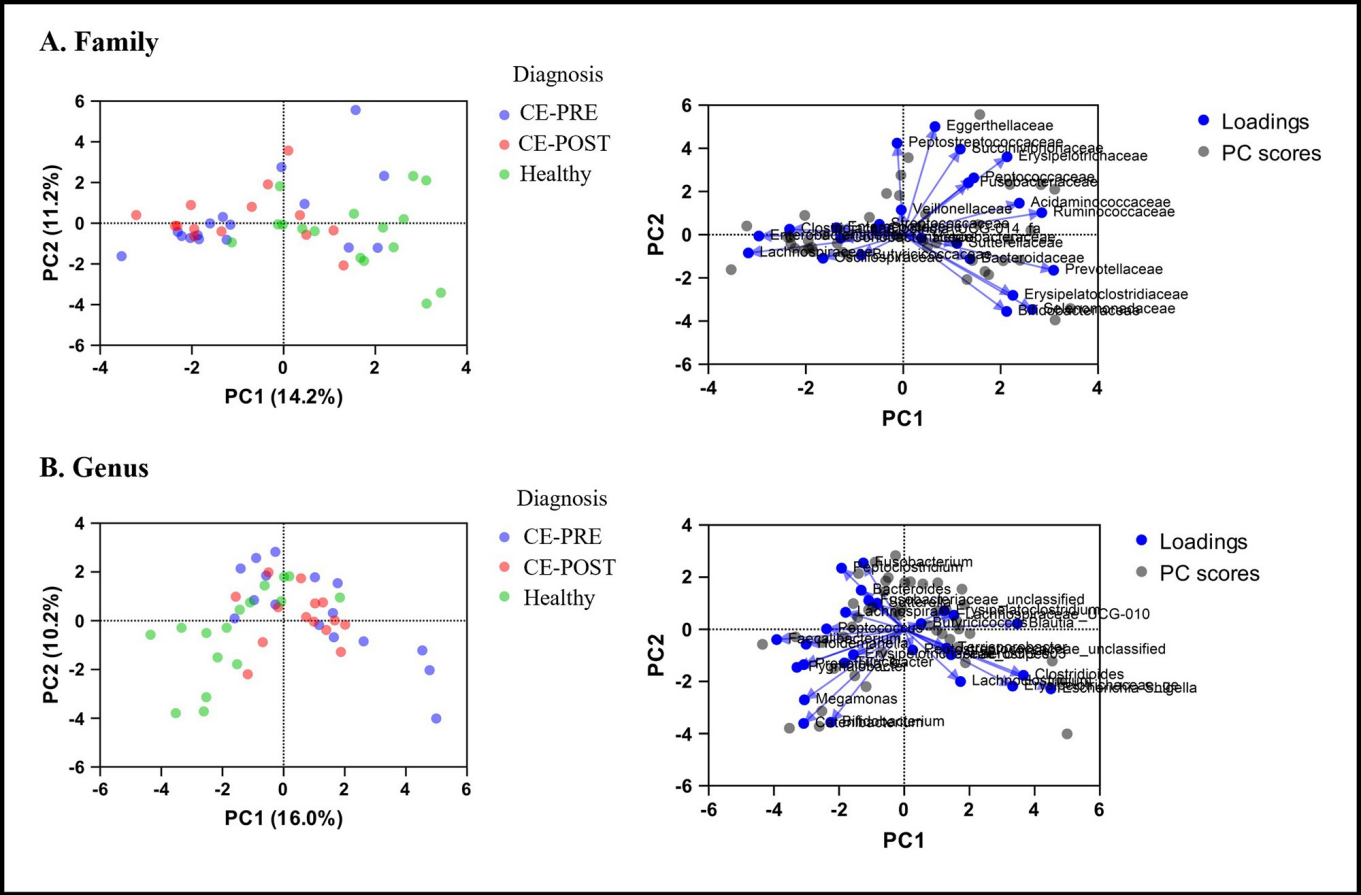
Principal component analysis (PCA) and biplots of fecal 16S rRNA gene sequencing in dogs with untreated chronic enteropathies (CE-PRE; blue), after 30 days of individualized chronic enteropathy treatment (CE-POST; red), and healthy dogs (green). Panel (A) represents Family level analysis. Panel (B) represents Genus level analysis. A least squares regression model performed on PCA with GLP-2 concentration as the outcome identified 6 families and 10 genera that positively contributed to GLP-2 variance. Nine genera were identified that negatively contributed to GLP-2 variance.

Individual families with significant positive contribution included Prevotellaceae, Ruminococcaceae, Erysipelotrichaceae, Acidaminococcaceae, Peptococcaceae, and Succinivibrionaceae. At the genus level (R^2^ = 0.276; F (2,43) = 8.190; *P* = 0.001), variance in plasma GLP-2 concentration was positively contributed to by relative abundance of *Prevotella*_9, *Megamonas*, *Catenibacterium*, *Faecalibacterium*, *Holdemanella*, *Turicibacter*, *Peptococcus*, *Erysipelotrichaceae*_UCG-003, *Pygmaiobacter*, and *Lachnospira*. Genera that negatively contributed to the variance in GLP-2 concentrations included *Blautia*, *Escherichia-Shigella*, *Terrisporobacter*, *Clostridioides*, *Erysipelatoclostridium*, *Anaerostipes*, *Butyricicoccus*, *Erysipelotrichaceae_ge*, and *Lachnospiraceae_UCG-010* (Tables 2 and 3; Fig 5).

**Table 2:** Bacterial OTU positively associated with plasma GLP-2 concentrations.

OTU	β [95% CI]	|t|	*P* value
**Family**			
Acidaminococcaceae	13972 [5887,22057]	3.493	0.001
Erysipelotrichaceae	2369 [663,4074]	2.807	0.008
Peptococcaceae	12369 [4816,19922]	3.310	0.002
Prevotellaceae	375.7 [130.6,620.8]	3.098	0.004
Ruminococcaceae	3109 [1557,4661]	4.048	<0.001
Succinivibrionaceae	17660 [1014,34306]	2.144	0.038
**Genera**			
*Catenibacterium*	1024 [136,1912]	2.326	0.025
*Erysipelotrichaceae_*UCG-003	9744 [3588,15901]	3.192	0.003
*Faecalibacterium*	1860 [932,2787]	4.044	<0.001
*Holdemanella*	1662 [821,2503]	3.981	<0.001
*Lachnospira*	28563 [8010,49117]	2.803	0.008
*Megamonas*	511 [132,889]	2.723	0.009
*Peptococcus*	4765 [2369,7161]	4.010	<0.001
*Prevotella_*9	226 [98,355]	3.569	<0.001
*Pygmaiobacter*	9022 [13937,50326]	3.561	<0.001
*Turicibacter*	4196 [1410,6983]	3.037	0.004

Beta coefficient and 95% confidence interval of OTUs (operational taxonomic units) positively contributing to the variance in plasma glucagon-like peptide-2 concentrations in 16 healthy dogs, 16 untreated CE dogs (CE-PRE) and 14 CE dogs after 30 days of treatment (CE-POST). Significance based on *P* <0.05.

^1^ CE = chronic enteropathy

**Table 3:** Bacterial OTU negatively associated with plasma GLP-2 concentrations.

OTU	Β [95% CI]	|t|	*P* value
**Genera**			
*Anaerostipes*	-926 [-1740,-112]	2.294	0.027
*Blautia*	-280 [-419,-140]	4.046	<0.001
*Butyricicoccus*	-3552 [-5798,-1306]	3.189	0.003
*Clostridioides*	-1854 [-3176,-532]	2.828	0.007
*Erysipelatoclostridium*	-1097 [-1892,-301]	2.779	0.008
*Erysipelotrichaceae_ge*	-38097 [-70684,-5509]	2.358	0.023
*Escherichia-Shigella*	-675 [-1169,-181]	2.755	0.009
*Lachnospiraceae_UCG-010*	-40468 [-64461,-16474]	3.401	0.002
*Terrisporobacter*	-610 [-1082,-139]	2.608	0.012

Beta coefficient and 95% confidence interval of OTUs (operational taxonomic units) negatively contributing to the variance in plasma glucagon-like peptide-2 concentrations in 16 healthy dogs, 16 untreated CE dogs (CE-PRE), and 14 CE dogs after 30 days of treatment (CE-POST). Significance based on *P* <0.05.

^1^ CE = chronic enteropathy

## Discussion

Through this study, we present the first association between plasma GLP-2 concentration and fecal microbiota populations in dogs with CE. We compared the fecal microbiome of dogs with CE prior to and following approximately one month of individualized treatment to that of healthy dogs. Through concurrent analysis of plasma GLP-2, we identified microbiota populations that contributed to variance in GLP-2 concentrations between CE dogs and HC. Results also highlighted limitations in commercially available assays to predict dysbiosis.

Consistent with previous studies [5–10], both the DI, and associated qPCR, and 16S V4 rRNA analyses highlighted differences in GI microbiota in CE dogs compared to HC. In contrast to previous work [32], however, the utility of the DI to accurately reflect dysbiosis in CE dogs and differences between CE dogs and HC was limited in this study. While there was no significant difference in DI index value between groups, numerically the median index value was lower (i.e., more normal) in HC dogs, and the lack of statistical difference was therefore likely reflected by small sample size and multiple comparisons. Interestingly, three HC dogs had a DI between 0–2, reflecting a grey zone or mild dysbiosis value, and one HC had DI >2, reflecting dysbiosis. None of those dogs had any abnormalities in individual taxa. When using the DI as a diagnostic for dysbiosis in CE dogs, only 5 out of 16 index values were elevated at baseline. When evaluating individual taxa, the decreased *Turicibacter* abundance at baseline in CE dogs is consistent with known disturbances in dogs with GI disease [9]. Notably, none of the individual taxa abundances significantly changed in CE dogs between baseline and recheck evaluation; although, *Blautia* tended to decrease at recheck, which contrasts with what one would expect for resolution of dysbiosis. Furthermore, 8 out of 14 index values were consistent with mild or overt dysbiosis at study reevaluation. The bacterial groups that contributed to the shifts in DI varied among individual dogs, which is likely why group-wide shifts in bacterial abundances were not appreciated. The minimal change or worsening in DI occurred despite the clinical improvement documented through the canine inflammatory bowel disease activity index (CIBDAI) and canine chronic enteropathy activity index (CCECAI) in most dogs [28]. This underscores the importance of considering factors that could impact the microbiome, such as antimicrobials and other medications used for CE treatment, in addition to GI disease status when utilizing the DI as a monitoring tool. Though, a normal DI in over 30% of CE dogs has been reported in previous studies, as well [5–7, 32, 33].

In contrast to the few microbiome differences detected between CE dogs and HC through DI analysis and qPCR, numerous shifts in bacterial taxa were noted in the CE-PRE group compared to HC through 16S V4 rRNA analysis, predominantly reflected in decreased OTU abundances. As only two OTUs differed between CE-POST and HC groups, 16S V4 rRNA analysis suggested an improvement in dysbiosis not reflected by DI analysis. 16S V4 gene sequencing identified significant decreases in *Faecalibacterium*, *Turicibacter*, and *Fusobacterium*; whereas only decreased *Turicibacter* abundance was identified via qPCR analysis. This likely reflects the inherent differences in methodology between qPCR assays and 16S V4 rRNA gene sequencing; though, differences related to the region of the 16S gene that is targeted should also be considered.

Importantly, and relevant to the results of this study, 16S V4 rRNA sequencing has advantages in identifying microbiota that contribute to disease manifestation, particularly in the early stages of exploring a disease process. Using 16S V4 rRNA sequencing, this study demonstrated positive associations on circulating GLP-2 concentrations with increasing relative abundance of 10 genera and 6 families. All 10 genera (*Catenibacterium*, *Erysipelotrichaceae*_UCG-003, *Faecalibacterium*, *Holdemanella*, *Lachnospira*, *Megamonas*, *Peptococcus*, *Prevotella*_9, *Pygmaiobacter*, *Turicibacter*) were significantly decreased in CE-PRE. A significant negative association with GLP-2 concentration was observed with abundance of 8 genera, including *Escherichia-Shigella*, *Clostridioides*, and *Anaerostipes*, the only 3 genera that were significantly increased in CE-PRE. These findings suggest that increases in plasma GLP-2 following CE treatment are associated with a trend toward normobiosis.

Worth noting is that all 5 of the bacterial groups that were both significantly decreased in dogs with CE-PRE and negatively associated with GLP-2 concentrations (*Blautia*, *Lachnospiraceae*, *Butyricicoccus*, *Erysipelatoclostridium*, and *Erysipelotrichaceae)* are butyrate-producing bacteria within the phylum Firmicutes [34, 35]. This was an unexpected finding given that butyrate strongly stimulates GLP-2 secretion [36]. As expected, other SCFA-producing bacteria, such as *Faecalibacteria*, *Turicibacter*, *Ruminoccaceae*, and *Lachnospira*, showed strong positive associations with GLP-2 concentrations and were significantly decreased in CE-PRE dogs. These findings suggest that the latter populations may more highly impact enteroendocrine responses in dogs compared to those in the above-mentioned butyrate-producing species. However, it is also possible that the specific metabolic by-products are more important for GLP-2 secretion than individual microbiota. Metabolome analyses for SCFA in conjunction with GLP-2 concentrations could more clearly define this relationship.

*Clostridium hiranonis*, currently known as *Clostridioides hiranonis*, is a bile acid converter commonly associated with diet-responsiveness in CE dogs [10, 37]. Although *C*. *hiranonis* was not significantly different between CE-PRE and CE-POST samples or between CE dogs and HC based on DI analysis, this was the most common bacterial species outside the normal relative abundance reference range in either CE-PRE (*n* = 4) or CE-POST (*n* = 6) fecal samples. Antimicrobial administration has been associated with decreased fecal secondary bile acids due to the high antimicrobial sensitivity of *C*. *hiranonis* [38, 39], and three dogs in our study received antimicrobials as part of their CE treatment [28]. All three dogs had normal *C*. *hiranonis* abundances at presentation but severely decreased concentrations at follow-up (range, 0.1–1.5 logDNA [reference interval 5.1–7.1]). As intraluminal bile acids stimulate GLP-2 secretion from L-cells [15], lack of *C*. *hiranonis* normalization could explain the lack of complete GLP-2 normalization in CE-POST dogs. This cannot be confirmed, however, as none of the OTU analyzed via qPCR were significantly associated with variance in GLP-2 concentrations among groups. Further, metabolome analysis for secondary bile acids would be needed to confirm this relationship in dogs.

Loss of the commensal *C*. *hiranonis* has been associated with increased pathogenic species such as *C*. *difficile*, *C*. *perfringens*, and *E*.*coli* [37]. Potentially consistent with this observation, 16S V4 rRNA gene sequencing revealed an increase in *Clostridioides* in CE-PRE dogs compared to controls, which resolved with treatment. Quantitative analysis for distinct bacterial species would be required to further define the populations within this genus that contributed to the change. *Escherichia-Shigella* was the only genus that remained significantly increased in CE-POST dogs. *Escherichia-Shigella* has been negatively associated with GLP-2 secretion in mouse models [40], consistent with our findings in dogs. Persistent elevation of *Escherichia-Shigella* in CE-POST dogs may partially explain failure of GLP-2 to normalize in these dogs despite significant improvement from pre-treatment GLP-2.

This study was limited by its small sample size, which was not recruited specifically for the objectives of this study, but rather to determine differences in GLP-2 concentrations among groups with the expectation that shifts in microbiota would be related to GLP-2 changes. Therefore, results may underestimate significant changes in the microbiome associated with disease, treatment, and circulating GLP-2. This may be particularly relevant where *P* values adjusted for multiple comparisons did not demonstrate a significant difference where a standard *P* value of <0.05 would have demonstrated a statistically significant difference (e.g., lack of statistical difference in *Blautia spp*. abundances between CE-PRE and HC). These analyses might demonstrate significant differences in a larger study population. Lack of standardized treatment may likewise differentially affect the microbiome, especially in the three patients that received antimicrobials, which had some of the highest increases in post-treatment DI, rather than normalization. As type and severity of GI disease influence fasting and post-prandial GLP-2 secretion patterns in humans with IBD [22–27], including a range of disease in our population, rather than focusing on dogs with ileal disease, may have limited the ability to observe relationships between microbiota and GLP-2 concentrations. However, dogs have a higher concentration of L-cells more proximally in their GI tract (i.e., jejunum) than other species; therefore, it may not be possible to extrapolate disease localization and impact on GLP-2 secretion among species. Inclusion of larger numbers of dogs with individual disease subsets, such as protein-losing enteropathies, and implementation of standardized treatments are considerations for future studies. However, this population of dogs more accurately represents those encountered in a clinical setting, making findings more applicable to the canine CE population as a whole. It is also possible that the duration of the study did not allow sufficient time for complete resolution of dysbiosis. One study showed that the microbiome of CE dogs still differed significantly from controls despite clinical remission after 8 weeks of treatment [7]. At a 1-year follow-up, no difference was observed between CE dogs and HC. Extending the follow-up time may result in a stronger correlation between dysbiosis and GLP-2 secretion, particularly as GLP-2 had also not normalized at the 30-day follow-up [28]. Finally, the methods used to assess the GI microbiome may have underrepresented certain bacteria that play an important role in GLP-2 secretion. For example, *Akkermansia muciniphila* is a mucin-degrading bacterium that resides in the mucus layer, making it unlikely to be accurately represented via fecal microbiome analysis. In mouse models, *A*. *muciniphila* has been shown to increase intestinal concentrations of 2-oleoglycerol, which stimulates GLP-2 secretion from L-cells [17, 18]. While *A muciniphila* has not been documented as a component of the normal canine GI microbiome, other mucin-degrading bacteria could contribute to this role in dogs. Despite its limitation in identifying these populations, fecal microbiome analysis has the benefit of being non-invasive and is a routinely accepted method of microbiome description in dogs. Lastly, microbiome studies vary in analytical approach. While we chose to evaluate microbiota clustering through OTUs and focus on abundance differences through Bray-Curtis dissimilarity analysis, assessments using amplicon sequence variants or phylogenetic relationships through UniFrac distance analyses could result in differences in diversity outcomes or identify functionally related microbiota groups of importance.

## Conclusions

Overall, this study demonstrated both positive and negative correlations between circulating GLP-2 concentrations and specific fecal OTUs in dogs. The association between increased plasma GLP-2 and normalization of dysbiosis lends additional support for the microbiome as a target for canine CE management. However, this study highlighted the limitations of commercially available assays for dysbiosis description. At the present time, their use for disease exploration in this area as an alternative to 16S V4 rRNA sequencing methods cannot be recommended. Future studies targeting the genera identified in this exploratory study, as well as metabolome analyses, are warranted. Correlating findings to assessment of mucosal barrier function would be particularly useful in determining a potential therapeutic benefit to CE dogs.

## Supporting information

S1 Table CE dog treatments at visit 2 (CE-POST). Medications, supplements, and/or dietary management provided to individual dogs for treatment of their chronic enteropathy (CE).
(DOCX)

S2 Table OTU group differences. Log fold change in OTU with significantly different abundances between healthy dogs and dogs with untreated chronic enteropathy.
(DOCX)

S1 Fig Analytical flowchart. (JPG)

S2 Fig Taxa identified through 16S V4 rRNA sequencing. Major bacterial A. Phyla B. Classes C. Orders D. Families E. Genus identified through 16S V4 rRNA sequencing and relative abundances in healthy dogs and dogs with chronic enteropathies prior to treatment (CE-PRE/visit 1) and after 30 days of individualized GI disease treatment (CE-POST/visit 2).
(JPG)

## Abbreviations

BCS: body condition score
CE: chronic enteropathy
CE-POST: chronic enteropathy post-treatment
CE-PRE: chronic enteropathy pre-treatment
DI: dysbiosis index
GI: gastrointestinal
GLP-2: glucagon-like peptide-2
HC: healthy control
OTU: operational taxonomic unit
PCoA: principal coordinate analysis
qPCR: quantitative PCR
SCFA: short chain fatty acid
UC: ulcerative colitis
