# Supplementary material for: Evaluation of the relationship between plasma glucagon-like peptide-2 and gastrointestinal dysbiosis in canine chronic enteropathies
Source: PLoS One. 2024 Jun 27;19(6):e0305711. doi: 10.1371/journal.pone.0305711 (PMC11210855; doi:10.1371/journal.pone.0305711)
Supplement: S1 Table — Medications, supplements, and/or dietary management provided to individual dogs for treatment of their chronic enteropathy (CE). (DOCX) [file pone.0305711.s001.docx]

**S1 Table. CE dog treatments at visit 2 (CE-POST)**

| **Dog Study Number** | **Therapeutic Diet** | **Medications** | **Supplements** |
| --- | --- | --- | --- |
| 1 | Hydrolyzed protein | Amoxicillin 23 mg/kg q12h,  bismuth subsalicylate 10 mg/kg q8h, metronidazole 20 mg/kg q12h |  |
| 6 | Novel protein | Clopidogrel 1.3 mg/kg q24h, prednisone 0.86 mg/kg q24h |  |
| 7 | Hydrolyzed protein |  |  |
| 8 | Hydrolyzed protein | Prednisone 1.5 mg/kg q24h |  |
| 9 | Hydrolyzed protein | Prednisone 1.3 mg/kg 12h |  |
| 11 | Hydrolyzed protein | Amoxicillin 22 mg/kg q12h, clarithromycin 7 mg/kg q12h, metronidazole 20 mg/kg q24h |  |
| 13 |  | Chlorambucil 0.22 mg/kg q24h, clopidogrel 3.5 mg/kg q24h, omeprazole 1 mg/kg q12h,  prednisone 0.72 mg.kg q12h | Cyanocobalamin SQ q7 days (dose not recorded in medical record) |
| 14 | Novel protein | Calcium carbonate 72 mg/kg q24h, prednisone 1.44 mg/kg q24h | Cyanocobalamin 500 µg (72 µg/kg) SQ q7 days |
| 15 | Easily digestible | Budesonide 0.15 mg/kg q24h |  |
| 16 | Hydrolyzed protein | Clopidogrel 2.5 mg/kg q24h, prednisone 1.3 mg/kg q12h | Folate 25 µg/kg q24h |
| 17 | Hydrolyzed protein |  | Cyanocobalamin 250 µg (54 µg/kg) SQ q7 days |
| 18 |  | Amoxicillin 25 mg/kg q12h, clarithromycin 15 mg/kg q12h, metronidazole 30 mg/kg q24h |  |
| 19 |  | Prednisone 2 mg/kg q24h |  |
| 21 | Easily digestible | Calcium carbonate 654 mg/kg q24h, clopidogrel 2.4 mg/kg q24h, prednisone 2 mg/kg q24h |  |
