# Supplementary material for: Evaluation of the relationship between plasma glucagon-like peptide-2 and gastrointestinal dysbiosis in canine chronic enteropathies
Source: PLoS One. 2024 Jun 27;19(6):e0305711. doi: 10.1371/journal.pone.0305711 (PMC11210855; doi:10.1371/journal.pone.0305711)
Supplement: S2 Table — Log fold change in OTU with significantly different abundances between healthy dogs and dogs with untreated chronic enteropathy. (DOCX) [file pone.0305711.s002.docx]

**S3 Table. OTU group differences**

|  | **Phylum** | **Family** | **Genus** | **log2Fold**  **Change** | **padj** |
| --- | --- | --- | --- | --- | --- |
| Otu0002 | Bacteroidota | Prevotellaceae | Prevotella_9 | -4.86165 | 0.01745 |
| Otu0005 | Firmicutes | Selenomonadaceae | Megamonas | -5.56881 | 0.00360 |
| Otu0010 | Proteobacteria | Enterobacteriaceae | Escherichia-Shigella | 4.65230 | 0.03380 |
| Otu0013 | Firmicutes | Erysipelatoclostridiaceae | Catenibacterium | -5.69952 | 0.00360 |
| Otu0018 | Firmicutes | Ruminococcaceae | Faecalibacterium | -4.56876 | 0.00983 |
| Otu0021 | Firmicutes | Erysipelotrichaceae | Holdemanella | -3.88205 | 0.04397 |
| Otu0028 | Bacteroidota | Bacteroidaceae | Bacteroides | -4.01161 | 0.01476 |
| Otu0035 | Firmicutes | Lachnospiraceae | Blautia | -4.13063 | 0.00519 |
| Otu0039 | Firmicutes | Peptostreptococcaceae | Clostridioides | 3.16738 | 0.02577 |
| Otu0043 | Bacteroidota | Bacteroidaceae | Bacteroides | -4.38139 | 0.00440 |
| Otu0045 | Firmicutes | Lachnospiraceae | Lachnoclostridium | -3.29657 | 0.00519 |
| Otu0046 | Proteobacteria | Sutterellaceae | Sutterella | -2.86676 | 0.04397 |
| Otu0047 | Firmicutes | Lachnospiraceae | Lachnoclostridium | -2.80997 | 0.04397 |
| Otu0057 | Firmicutes | Erysipelatoclostridiaceae | Erysipelatoclostridium | -2.79592 | 0.03000 |
| Otu0060 | Firmicutes | Erysipelotrichaceae | Turicibacter | -3.80434 | 0.01745 |
| Otu0062 | Bacteroidota | Bacteroidaceae | Bacteroides | -2.77971 | 0.03000 |
| Otu0069 | Firmicutes | Peptococcaceae | Peptococcus | -2.49254 | 0.02512 |
| Otu0080 | Campylobacterota | Helicobacteraceae | Helicobacter | -2.70932 | 0.02364 |
| Otu0082 | Bacteroidota | Prevotellaceae | Prevotella_9 | -2.45138 | 0.02176 |
| Otu0085 | Firmicutes | Lachnospiraceae | Anaerostipes | 2.62863 | 0.03000 |
| Otu0088 | Bacteroidota | Prevotellaceae | Prevotella_9 | -2.53406 | 0.02520 |
| Otu0089 | Firmicutes | Erysipelatoclostridiaceae | Erysipelotrichaceae_  UCG-003 | -2.82638 | 0.00519 |
| Otu0093 | Bacteroidota | Prevotellaceae | Prevotella_9 | -2.50564 | 0.02176 |
| Otu0095 | Firmicutes | Ruminococcaceae | Pygmaiobacter | -3.50075 | 0.00015 |
| Otu0105 | Bacteroidota | Prevotellaceae | Prevotella_9 | -2.85483 | 0.00694 |
| Otu0117 | Firmicutes | Peptostreptococcaceae | Peptoclostridium | -1.95094 | 0.02512 |
| Otu0118 | Firmicutes | Selenomonadaceae | Megamonas | -2.71934 | 0.00440 |
| Otu0119 | Firmicutes | Selenomonadaceae | Megamonas | -1.82456 | 0.04058 |
| Otu0132 | Firmicutes | Peptostreptococcaceae | Peptoclostridium | -2.57620 | 0.00694 |
| Otu0137 | Firmicutes | Butyricicoccaceae | Butyricicoccus | -2.05909 | 0.00360 |
| Otu0142 | Fusobacteriota | Fusobacteriaceae | Fusobacterium | -1.78495 | 0.04058 |
| Otu0150 | Firmicutes | Peptostreptococcaceae | Peptoclostridium | -2.35376 | 0.00360 |
| Otu0157 | Firmicutes | Ruminococcaceae | Faecalibacterium | -2.02378 | 0.01758 |
| Otu0161 | Firmicutes | Selenomonadaceae | Megamonas | -2.24503 | 0.00313 |
| Otu0169 | Firmicutes | Selenomonadaceae | Megamonas | -1.66821 | 0.00519 |
| Otu0183 | Actinobacteriota | Coriobacteriaceae | Collinsella | -1.50349 | 0.04840 |
| Otu0184 | Bacteroidota | Bacteroidaceae | Bacteroides | -1.22203 | 0.03693 |
| Otu0198 | Firmicutes | Lachnospiraceae | Blautia | -1.84948 | 0.01986 |
| Otu0200 | Firmicutes | Erysipelatoclostridiaceae | Catenibacterium | -2.60553 | 0.00001 |
| Otu0211 | Firmicutes | Ruminococcaceae | Faecalibacterium | -1.26875 | 0.04245 |
| Otu0212 | Firmicutes | Erysipelatoclostridiaceae | Catenibacterium | -1.38336 | 0.02577 |
| Otu0231 | Firmicutes | Erysipelatoclostridiaceae | Catenibacterium | -1.78888 | 0.00030 |
| Otu0232 | Firmicutes | Lachnospiraceae | Blautia | -1.69662 | 0.00262 |
| Otu0245 | Firmicutes | Peptostreptococcaceae | Peptoclostridium | -1.41944 | 0.00385 |
| Otu0246 | Bacteroidota | Prevotellaceae | Prevotella_9 | -2.17361 | 0.00014 |
| Otu0259 | Firmicutes | Lachnospiraceae | Blautia | -1.39886 | 0.02972 |
| Otu0268 | Firmicutes | Lachnospiraceae | Lachnospiraceae_  unclassified | -1.93798 | 0.00519 |
