# Supplementary figures and images for: Evaluation of the relationship between plasma glucagon-like peptide-2 and gastrointestinal dysbiosis in canine chronic enteropathies
Source: PLoS One. 2024 Jun 27;19(6):e0305711. doi: 10.1371/journal.pone.0305711 (PMC11210855; doi:10.1371/journal.pone.0305711)

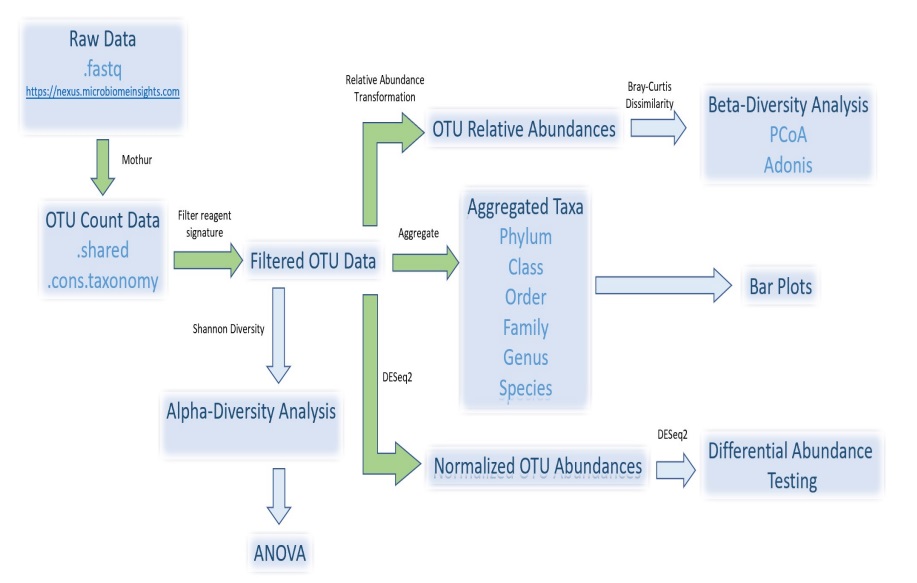

Supplement: S1 Fig — (JPG) [file pone.0305711.s003.jpg]

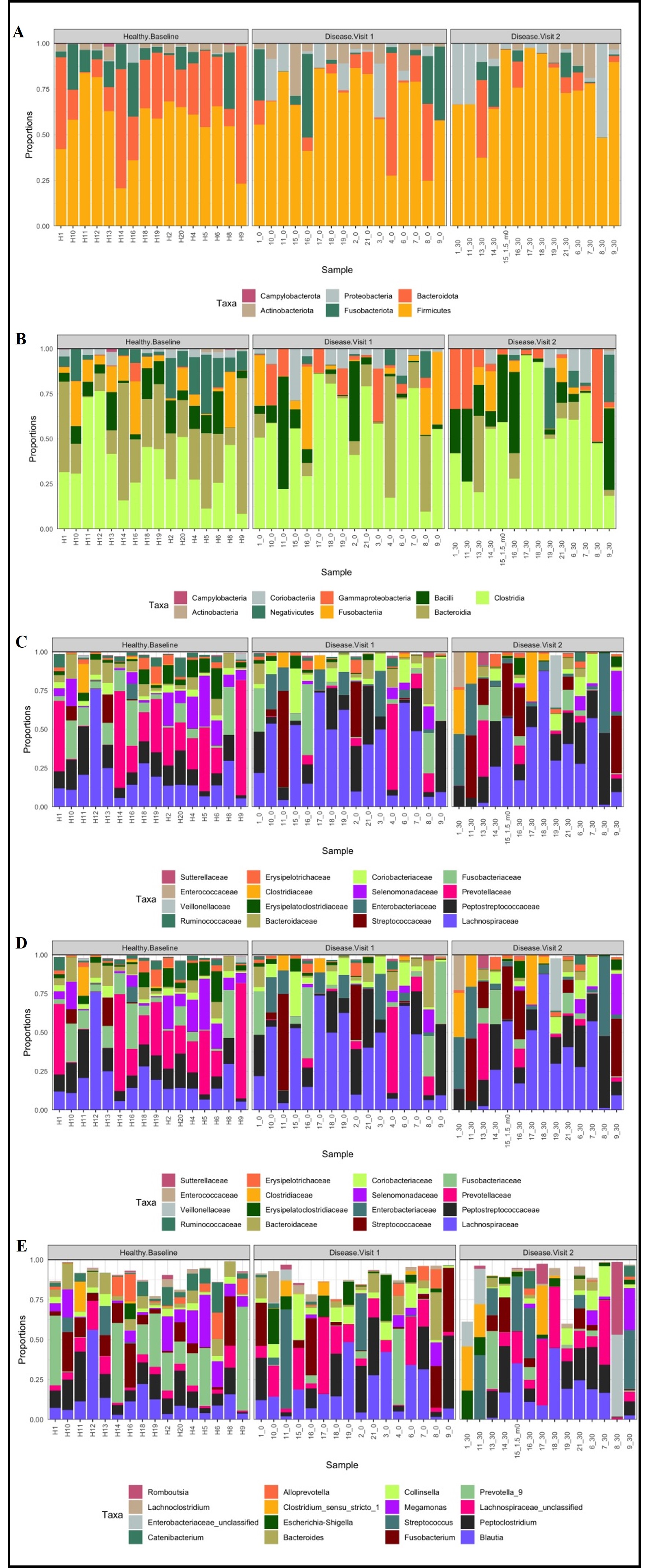

Supplement: S2 Fig — Major bacterial A. Phyla B. Classes C. Orders D. Families E. Genus identified through 16S V4 rRNA sequencing and relative abundances in healthy dogs and dogs with chronic enteropathies prior to treatment (CE-PRE/visit 1) and after 30 days of individualized GI disease treatment (CE-POST/visit 2). (JPG) [file pone.0305711.s004.jpg]
